# Overdose Deaths Involving Fentanyl and Fentanyl Analogs — New York City, 2000–2017

**DOI:** 10.15585/mmwr.mm6802a3

**Published:** 2019-01-18

**Authors:** Cody Colon-Berezin, Michelle L. Nolan, Jaclyn Blachman-Forshay, Denise Paone

**Affiliations:** 1 Bureau of Alcohol and Drug Use Prevention, Care, and Treatment, New York City Department of Health and Mental Hygiene, New York City, New York.

Unintentional drug overdose deaths have climbed to record high levels, claiming approximately 70,000 lives in the United States in 2017 alone (*1*). The emergence of illicitly manufactured fentanyl* (a synthetic, short-acting opioid with 50–100 times the potency of morphine) mixed into heroin, cocaine, and counterfeit pills, with or without the users’ knowledge, has increased the risk for fatal overdose (*2*,*3*). The New York City (NYC) Department of Health and Mental Hygiene (DOHMH) conducts routine overdose mortality surveillance by linking death certificates with toxicology findings from the NYC Office of the Chief Medical Examiner (OCME). A 55% increase in the rate of fatal drug overdose in NYC was observed from 2015 to 2017, resulting in the highest number of overdose deaths recorded since systematic reporting began in 2000. Toxicology data indicate that this unprecedented increase in overdose deaths is attributable to fentanyl. Early identification of increased fentanyl involvement enabled DOHMH to respond rapidly to the opioid overdose epidemic by increasing awareness of the risks associated with fentanyl and developing effective risk reduction messaging. These results strongly suggest that, wherever possible, jurisdictions should consider integrating toxicology findings into routine overdose surveillance and work with local medical examiners or coroners to include fentanyl in the literal text on death certificates.

Since 2013, illicitly manufactured fentanyl has been involved in a growing number of overdose deaths nationally (*3*,*4*) and in NYC and has been represented increasingly in seizures of synthetic opioids (*5*,*6*). The increased presence of fentanyl in the illicit drug market has implications for overdose prevention efforts; however, national reporting on the presence of fentanyl in overdose deaths is limited by the lack of standardized toxicology testing for fentanyl and the inconsistent listing of fentanyl as a cause of death on death certificates, resulting in underreporting of fentanyl involvement in fatal overdoses. Nationally, reporting on drugs involved in overdose deaths relies on death certificate data; despite local efforts to improve drug reporting on death certificates, at least 15% of overdose deaths do not specify any drugs (*7*). Thus, drug-specific data continue to be underreported, making it difficult to quantify the role of fentanyl in increasing overdose death rates.

For this analysis, DOHMH defined a death as an unintentional drug overdose if the medical examiner determined the manner of death to be accidental and the underlying or multiple-cause code was assigned an *International Classification of Diseases, Tenth Revision* code of X40–X44 (accidental overdose of drugs), F11–F16, or F18–F19 (excluding F-codes with 0.2 or 0.6 third digit).^†^ Toxicology findings were abstracted from OCME files and were used to classify overdose deaths according to the substances involved. Although OCME conducted fentanyl testing of all overdose cases during 2000–2012, universal testing for fentanyl was suspended during late 2013–July 2016, and the proportion of deaths tested during this time is unknown. However, despite inconsistent testing, in 2015 the proportion of all overdose deaths where fentanyl was detected exceeded that during the period of known universal fentanyl testing.

Unintentional drug overdose deaths were dichotomized according to whether or not any fentanyl was detected. The proportions of overdose deaths that involved fentanyl, overall and by other drug type involved, were calculated. Age-adjusted person-time rates were calculated by year using 2000–2016^§^ NYC population estimates adjusted to the U.S. Census 2000 projected population. Changes in rates were tested using z-tests and 95% confidence intervals; comparisons were based on the gamma confidence interval distribution.

Among 10,673 fatal overdoses in NYC during 2000–2014, a total of 7,822 (73%) involved an opioid. Fentanyl was determined to be involved in 246 of these deaths (i.e., 2% of all overdose deaths or 3% of deaths involving an opioid) (Figure). Beginning in 2015, the percentage of fentanyl-involved overdose deaths increased sharply; in 2016, 624 (44%) of 1,425 drug overdose deaths involved fentanyl, and in 2017, 842 (57%) of 1,487 overdose deaths involved fentanyl.

**FIGURE:**
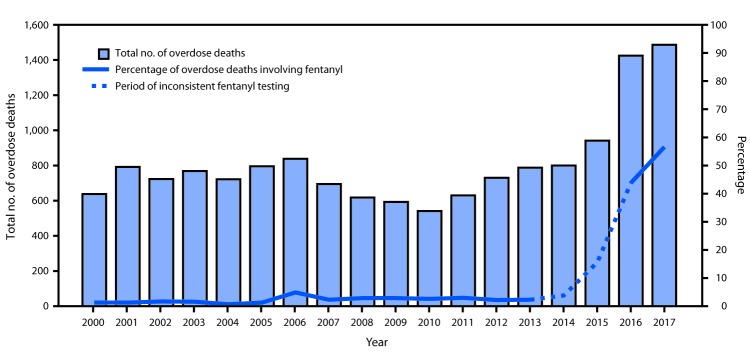
Number of overdose deaths and percentage of overdose deaths involving fentanyl* — New York City, 2000–2017 * Universal testing for fentanyl was stopped sometime during 2013 and restarted on July 1, 2016; fentanyl data during 2013–2016 were obtained from the Office of the Chief Medical Examiner but are known to be incomplete.

From 2014 to 2017, the rate of fentanyl-involved overdose deaths in NYC increased almost 3,000%, from 0.4 per 100,000 to 12.1. This trend is driving the overall increase in the rate of overdose deaths in NYC, which rose 81% during the same period, from 11.7 per 100,000 in 2014 to 21.2 in 2017, the highest rate since tracking of overdose deaths using this methodology began in 2000. In 2017, 531 (69%) heroin-involved deaths and 387 (53%) cocaine-involved overdose deaths also involved fentanyl (Table). Fentanyl also was involved in 146 (39%) deaths that involved cocaine but not heroin.

**TABLE:** Drug overdose deaths involving fentanyl, by selected drug(s) of involvement — New York City, 2017

Drugs	No. of overdose deaths	No. of overdose deaths with fentanyl detected (%)
**Total**	**1,487**	**842 (57)**
Heroin*	771	531 (69)
Cocaine*	732	387 (53)
Cocaine without heroin	379	146 (39)

*Drug categories are not mutually exclusive; includes deaths involving both heroin and cocaine.

## Discussion

DOHMH first detected an increase in fentanyl-involved overdose deaths in October 2015. Universal testing for the presence of fentanyl by OCME was suspended from 2013 through mid-2016, which made interpreting the significance of the observed increase a challenge. Working with DOHMH, OCME reinstituted universal testing for fentanyl by July 1, 2016. Despite the absence of systematic testing, DOHMH released a health advisory^¶^ in April 2016 confirming an increase in the rate of fentanyl-involved deaths, compared with previous periods of known universal testing (2000–2012). By the end of 2017, NYC recorded the highest number (1,487) and rate (21.2 per 100,000) of overdose deaths since tracking of overdose deaths using this methodology began in 2000 (*8*). From 2012, the last year of universal testing for fentanyl, to 2017, the first full year of resumed universal testing, the percentage of overdose deaths involving fentanyl increased from 2% (16 of 730) to 57% (842 of 1,487), suggesting that fentanyl was driving the observed increase in drug overdose deaths.

Without monthly surveillance of overdose deaths, timely detection of the emergent spike in fentanyl-involved deaths would not have been possible. The subsequent implementation of routine, comprehensive toxicology testing was critical to prospectively quantifying the extent of fentanyl’s presence in fatal overdoses. Whereas the use of toxicology data are central to overdose reporting in NYC, not all jurisdictions have the capacity to conduct toxicology testing on all suspected overdose cases or to abstract this information from medical examiner files. Health authorities without the capacity to abstract and report toxicology data can work with medical examiners to ensure drug-specific language is included in the cause of death fields on death certificates, which can improve the completeness of overdose reporting and tracking nationally. DOHMH has worked closely with OCME to ensure that suspected overdose cases are routinely tested for fentanyl, and that specific drugs (i.e., “fentanyl,” instead of “opioid”) involved in overdose deaths are listed in the literal text on death certificates, thus improving the quality of NYC overdose data (*9*).

The emergence of fentanyl as a factor in approximately half of the overdose deaths in NYC by 2016 necessitated novel and rapid strategies for disseminating information on reducing harms. After releasing routine health advisory notices in April and again in October 2016** to clinicians and health care providers citywide, DOHMH determined that more targeted communication concerning the risks associated with fentanyl was warranted. DOHMH conducted neighborhood-level outreach to syringe services programs and other programs that work with persons who inject drugs, advising them to 1) avoid using alone; 2) start with a small amount; 3) carry naloxone; and 4) avoid mixing drugs. Rapid responders were deployed to neighborhoods with higher rates of fentanyl-involved deaths to distribute educational flyers and fact sheets. This strategy was crucial to promoting public awareness of fentanyl in a timely manner.

As states expand overdose prevention efforts, the experience of NYC illustrates the importance of robust overdose surveillance, particularly in jurisdictions experiencing large increases in overdose deaths (*10*). Because of the high risk for overdose associated with using fentanyl, timely detection of fentanyl-involved deaths at the local level is critical (*10*). Incorporating systematic testing for fentanyl into overdose death investigations could provide vital information on underlying mortality causes and facilitate implementation of targeted overdose prevention education.

The findings in this report are subject to at least two limitations. First, universal testing for fentanyl was performed on all suspected overdose cases during 2000–2012 but was suspended late in 2013. In response to the detected increase in fentanyl involvement, OCME resumed universal testing for fentanyl on July 1, 2016. Therefore, fentanyl-involved overdose deaths might be underrepresented in the data during 2013–2016. Second, whereas fentanyl analogs clearly are illicitly manufactured, DOHMH is not able to distinguish deaths attributable to illicit fentanyl from those involving pharmaceutical fentanyl. However, analysis of NYC and national Prescription Drug Monitoring Program data indicate that while fentanyl seizures by law enforcement have risen sharply, rates of fentanyl prescription have remained stable (*4*). It is therefore likely that illicitly manufactured fentanyl, not pharmaceutical fentanyl, is driving NYC’s increase in drug overdose mortality.

The trends seen in NYC reflect the broader impact of fentanyl on rates of overdose deaths across the country, with the rate of overdose deaths involving synthetic opioids increasing by approximately 45% nationally from 2016 to 2017 (*1*). Addressing the fentanyl-driven overdose epidemic requires the coordinated efforts of public health authorities and medical examiners to systematically identify and list fentanyl in fatal overdose cases, to the extent possible. Access to drug-specific data can help target interventions and monitor their effectiveness. These measures will help direct the distribution of resources and the implementation of critical public health responses.

**Summary**

**What is already known about this topic?**

During 1999–2017, the rate of drug overdose deaths nationally approximately tripled; approximately 70,000 overdose deaths occurred nationally in 2017, with nearly 68% involving an opioid.

**What is added by this report?**

Using toxicology data, New York City identified fentanyl in 2% of drug overdose deaths during 2000–2012. By 2017, fentanyl was involved in 57% of all drug overdose deaths in New York City.

**What are the implications for public health practice?**

Universal fentanyl testing by local medical examiners and inclusion of drug-specific language on death certificates can aid surveillance and address the role of fentanyl in drug overdoses. Community-level educational outreach is indicated when an increase in fentanyl involvement is detected.

## Footnotes

* In this report, “fentanyl” refers to both the pharmacologic compound and any of its analogs.

† Disorders related to the use of opioids; cannabis; sedatives, hypnotics, or anxiolytics; cocaine; stimulants, hallucinogens; inhalants; or other psychoactive substances (excluding F-codes with 0.2 or 0.6 as the third digit, which specify a substance dependence or amnesic syndrome, respectively). DOHMH performs a secondary review on OCME case that have been assigned an X or F code because some F-coded cases involve acute drug intoxication. This review also facilitates reporting of toxicology involved.

§ DOHMH population estimates are modified from U.S. Census Bureau intercensal estimates.

¶ https://www1.nyc.gov/assets/doh/downloads/pdf/han/advisory/fentanyl-overdose.pdf.

** https://www1.nyc.gov/assets/doh/downloads/pdf/han/advisory/fentanyl-advisory.pdf.
